# Competency Profile for Primary Health Care Managers in Chile: Mixed-Methods Validation Through Expert Judgment

**DOI:** 10.3390/healthcare13243277

**Published:** 2025-12-13

**Authors:** Katherine Soto-Schulz, Raúl Herrera-Echenique, Nuria Pérez-Romero

**Affiliations:** 1 Exercise and Rehabilitation Sciences Institute, Postgraduate, Faculty of Rehabilitation Sciences, Universidad Andres Bello, Santiago de Chile 7591538, Chile; katherine.soto@unab.cl; 2 Programa de Doctorado en Derecho y Administración de Empresas, Universidad de Lleida, 25001 Lleida, Spain; 3 Facultad de Comunicación, Escuela de Periodismo, Universidad de Artes, Ciencias y Comunicación (UNIACC), Santiago de Chile 7501331, Chile; raul.herrera@uniacc.cl

**Keywords:** leadership, primary health care, professional development, health management, professional competence

## Abstract

**Background/Objectives**: Strengthening management competencies are essential to ensure effective and equitable Primary Health Care (PHC) systems. Emerging perspectives suggest that effective leadership in PHC could benefit from integrating cognitive, emotional, and social competencies. Although there is existing evidence on the required competencies worldwide and the characterization of PHC managers in Chile, no study has yet developed or validated a specific competency profile for these positions. This study developed and validated a competency profile for PHC managers to provide an evidence-based and context-specific tool for leadership, training, and evaluation. **Methods**: A mixed-method observational study based on expert judgment was conducted across three Chilean macrozones (northern, central, and southern), involving 36 professionals with recognized experience in PHC management identified through direct contact and snowball sampling. Quantitative validation through the Content Validity Index (CVI) confirmed high expert agreement (CVI ≥ 0.90), while thematic analysis of qualitative responses led to the inclusion of new areas. **Results**: The final profile comprises 47 competencies organized into knowledge, skills, and attitudes, emphasizing executive functions and social cognition and incorporating emerging domains such as emotional intelligence and institutional support. Beyond managerial relevance, the profile offers a structured framework for designing, implementing, and evaluating competency-based education and training in health sciences. **Conclusions**: These results also support leadership development and performance assessment, providing an evidence-based reference for professional training initiatives in PHC. This profile emphasizes that leadership development should not be limited to technical competencies, but also encompasses emotional, cognitive, and social dimensions essential for effective performance.

## 1. Introduction

Primary Health Care (PHC) is a strategic and essential approach to advancing towards universal health coverage, promoting equitable access to services for individuals and communities, as well as strengthening the resilience of health systems [1]. They also provide the operational context for leadership in complex and high-pressure environments. PHC managers must lead multidisciplinary teams, coordinate diverse services, and respond to changing social and institutional conditions. Beyond technical and procedural knowledge, affective and cognitive capacities could have a critical role in sustaining team cohesion, preventing burnout, and fostering resilient work environments. Effective leadership involves the integration of executive, emotional, and social brain networks [2,3]. These demands become even more relevant in PHC, where leaders must operate in settings marked by high social diversity, continuous change, and significant systemic pressures [4]. In this context, PHC seeks to ensure the highest possible level of health and well-being throughout the life course, ranging from health promotion and disease prevention to treatment, rehabilitation and palliative care [5].

Currently, PHC, considered the gateway to the health system [6], faces significant gaps: insufficient financing [7], fragmentation and segmentation of health systems [8], high levels of social inequality and precarious living conditions [9], an increase in the migrant population [10], and the need to strengthen human resources and health information systems [7]. These conditions affect timely and adequate access to health services, negatively impacting health outcomes, especially among the most vulnerable populations [11]. In addition, low pay, lack of professional autonomy and job instability contribute to demotivation among teams and affect the quality of care [12]. In the Chilean context, the current demands of PHC managers focus on strengthening the cultural competence of personnel in the face of growing diversity, improving inter-institutional coordination and continuity of care within a still-fragmented system, advancing toward truly comprehensive and community-oriented care, and addressing the historical weaknesses of health workforce planning, particularly the shortage and turnover of professionals, insufficient training in relevant competencies, and the need to effectively articulate actors such as universities, health services, and national authorities; all of this within a context in which reforms have prioritised financing and access, leaving strategic health workforce planning lagging behind [13,14].

Strengthening PHC requires managers who can lead complex processes and drive organizational transformations aimed at ensuring the quality, continuity and relevance of services [15]. Adequate management and strong leadership are essential for effective service delivery, as leaders not only provide a clear and guiding vision for the organization, but also mobilize, inspire and motivate teams [16]. The literature highlights that primary health care managers must have training in health, knowledge of care networks, information management and the use of management indicators, and preparation in administration and human resources [17]. Essential skills include leadership, planning, negotiation, problem solving, and team building [18,19,20], while in terms of attitude, autonomy, flexibility [19,21], proactivity [15], and ethical and socially responsible behaviour [20,22] are valued. These competencies, in addition to defining performance standards, can be considered for the design of competency-based education programs for Primary Health Care professionals. Strengthening these managerial competencies is further supported by evidence showing that leadership commitment and an enabling organizational culture are essential for effective, evidence-informed decision-making in healthcare systems [23].

Beyond these operational requirements, several leadership theories offer conceptual foundations that help explain how PHC managers can navigate complex and rapidly evolving health systems [24]. Transformational leadership emphasizes vision, inspiration, and the capacity to mobilize teams toward shared goals, elements that align with the relational and motivational demands of PHC management. Similarly, complex adaptive leadership highlights the need for flexibility, sensemaking, and adaptive responses within dynamic and resource-constrained environments. In the field of healthcare management, managerial effectiveness highlights the need for leadership that combines judgment and skill [16,25]. Models of excellence in quality management and principles of hospital administration require the integration of strategic (setting direction and vision), interpersonal (emotional intelligence and user orientation) and analytical/operational (responding to changing clinical priorities and setting high performance expectations) areas. This is essential to achieving a balance between administration and care, and between budgetary efficiency and service quality [16,24].

Implementing competency profiles is key to strengthening health systems, but their application must be contextualized to diverse institutional and territorial realities [26]. Their effectiveness depends, among other factors, on solid governance, access to adequate resources, and evidence-based approaches aligned with health objectives and environmental conditions [27]. In this sense, competency profiles guide professional development and strategic planning for the health workforce [28]. In Chile, research on PHC has identified key areas associated with both direct care and health centre management, highlighting the importance of integrating clinical skills with leadership, planning and administrative capabilities [29]. Although some primary care management positions are sometimes influenced by factors beyond competency criteria, such as staff rotation processes or organisational and political considerations [30,31], a defined competency profile remains essential to guide development, identify gaps, and ensure performance aligned with system needs.

This evidence underlines the need to define PHC management requirements suited to national and territorial contexts, with validation ensuring relevance, local alignment, and adaptability to other locally managed health systems. In addition, the validation of this profile may contribute to training processes aimed at strengthening leadership and management competencies among Primary Health Care professionals. The aim is to propose and validate a competency profile for PHC managers in Chile.

## 2. Materials and Methods

### 2.1. Design

A mixed-method approach [32] and observational, descriptive, cross-sectional design were used, with validation through expert judgement. The STROBE guidelines were used. Based on a previous review [17] and public recruitment calls, this study validates the proposed competency profile for PHC managers.

### 2.2. Participants

In the first stage, experts in the field were directly contacted. Experts were defined in accordance with previous Chilean studies, which characterize PHC experts as professionals with extensive experience in primary care, proven leadership trajectories, or academic contribution in the field [29,33]. Subsequently, these experts were invited to disseminate the survey among other professionals who met the predefined inclusion criteria, through non-probability convenience and snowball sampling [34]. Considering Chile’s geographical and population diversity, participants from the north, centre, and south were invited if they met at least one of the following criteria:

– Professionals who are currently serving as PHC directors for at least two years or who have held this position within the past five years.
– Professionals currently working in PHC roles, with a minimum of five years’ experience.
– Academics or researchers whose teaching or publications focused on PHC.

#### Instruments

A Google Form was used to collect the information, which included the following sections: (1) Informed consent; (2) Sociodemographic data: age, gender, highest education level (bachelor’s/diploma/master’s/doctorate/post doctorate), geographical area (North/Centre/South), years of experience in PHC, current role (management/care/teaching/research), years in current role and institution and participation in training related to professional competencies (yes/no/don’t remember); and (3) expert validation of competency profile [17] including multiple-choice items where each competency (knowledge, skill, attitude) was defined and rated from 1 to 4 (not relevant to highly relevant). Open-ended questions allowed experts to suggest additional relevant competencies. This is not a scale but a competency profile, organized into knowledge, skills, and attitudes rather than items or response dimensions.

### 2.3. Procedure

Primary health care professionals were initially contacted to help disseminate the form to eligible experts, using personal networks and social media. Eligible participants were then invited by email to complete the online form. Responses were collected between 28 February and 30 April 2025. Data were subsequently consolidated and analysed. The final profile was built by integrating all data, while geographic stratification was used only in descriptive analysis to ensure transparency and bias control.

### 2.4. Data Analysis

The Shapiro–Wilk test was used to assess data normality, appropriate for small to medium samples (*p* = 0.05). For normally distributed data, mean and standard deviation were reported; otherwise, median and interquartile range were used. Categorical variables were summarized with frequency tables. The separation of descriptive data by northern, central, and southern macrozones was performed to reflect the heterogeneity of Chile’s PHC system, which varies in organizational structures, managerial practices, and contextual characteristics across territories. This disaggregation was included to enhance transparency and allow readers to interpret potential regional nuances if relevant. Analyses were conducted using SPSS 23 and Excel 365.

Content validity was assessed with the Content Validity Index (CVI), using a 4-point scale to avoid neutral responses (used too by de Souza et al. (2017) [35]). This index was calculated as: IVC = number of responses 3 or 4/total number of responses, with an accepted level of at least 0.80 and, preferably, above 0.90. The CVI was computed both for the overall competency and for each individual competency within the profile.

Qualitative data derived from the open-ended responses included in the survey were analysed using a reflexive thematic analysis approach. Two researchers independently reviewed the comments and then met to discuss their interpretations. Through consensus meetings, the contributions were categorized, reorganized, or incorporated into new competencies as appropriate. No qualitative analysis software was used; all responses and coding decisions were systematically organized in spreadsheets. The dataset consisted of three open-ended questions asking participants to indicate which competencies they believed were missing in each category (knowledge, skills, and attitudes). Trustworthiness was strengthened through independent coding, iterative comparison between coders, and the resolution of discrepancies through discussion.

### 2.5. Ethical Considerations

This study was conducted in accordance with the ethical principles of the Belmont Report [36]. Participation was voluntary, without incentives, subject to informed consent, and with the right to withdraw from the study at any time. The protocol was approved by the Scientific Ethics Committee of San Juan de Dios Hospital in session No. 245, on 29 January 2025, and protocol minutes 334, version 1.

## 3. Results

### 3.1. Participants

**Figure 1:**
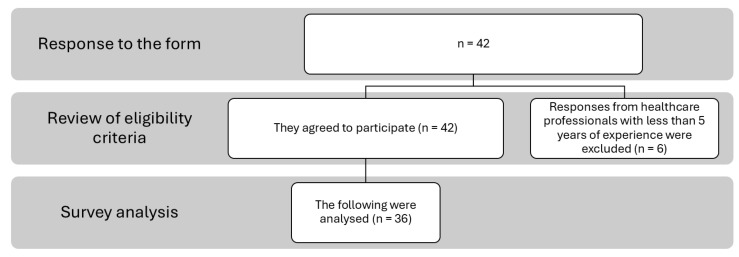
Flowchart of the selection process.

Of the initial 42 experts, six responses were excluded due to less than five years of experience in primary health care. Consequently, 36 responses were analysed (Figure 1).

### 3.2. Descriptive Analysis and Normality

The descriptive analysis was carried out by geographical areas (north, centre and south). Age in the north, years of experience in PHC the north and centre, and years of experience in current position in centre were reported as medians; the rest were reported as means according to normality analyses (see Table 1).

Participants were predominantly women across all macro-zones, and most had substantial professional experience in PHC, with average trajectories exceeding nine years. Of the 36 participants, 5 were from the north, 26 from the centre, and 5 from the south. Median age in the north was 35 years (IQR: 32–35); mean age in the centre was 39.89 (SD = 7.20), and in the south, 37.4 (SD = 4.62). Women predominated in all zones: 80% (north), 59.69% (centre), and 60% (south) (Table 2).

Professional roles also differed by region: the north and south showed a stronger presence of care-oriented professionals, whereas the centre exhibited a more heterogeneous mix, including management, teaching, and research roles. Professional roles varied: in the north, 60% worked in a care role in primary care and 40% combined a care role in primary care and management. In the centre, 34.62% worked in a care role in primary care, 30.77% were management, 23.08% combined a care role in primary care and teaching, and 3.85% combined a care role in primary care, teaching and research. In the south, 80% worked in care roles and 20% in management.

Educational attainment was generally high, particularly in the centre and south, where master’s degrees were common, while diplomas were more frequent in the north. A master’s degree was the most frequent qualification in the centre (50%) and south (60%). Diplomas were common in all regions, especially the north (60%). Only one participant had a doctorate (3.85%), in the centre. Bachelor’s degrees appeared mainly in the north (20%). The median PHC experience was 9 years (north), 12 (centre), and the mean 12.80 (south). Experience in the current role was 9 years (north), 6.50 years (median, centre), and 9.80 (south). The mean of institutional experience was between 9.20 (north), 10.15 (centre) and 9.80 (south). Training in competencies was reported by 60% (north), 92.31% (centre), and 100% (south); and 40% (north), 7.69% (centre) and 0% (south) had no training, they did not remember it or could not recall.

### 3.3. Content Validity

None of the CVI was lower than 0.92 (Table 3), so all remained within the profile. The proportion of competencies with CVI = 1 in each category was 83% for knowledge, 35% for skills and 79% for attitudes).

### 3.4. Qualitative Analysis

The qualitative analysis identified elements that complement, reorganize or refine the profile (Appendix A). The contributions were analysed and categorized (Appendix C), determining the final competency profile (Appendix B). Analysis of the qualitative items showed that 33.33% (12 responses) for knowledge, 63.89% (23 responses) for skills, and 72.22% (26 responses) for attitudes indicated no need to add anything; therefore, qualitative additions were provided in 24 responses for knowledge, 13 for skills, and 10 for attitudes. Of the total 36 participants, 12 responded “no need to add anything” to all three qualitative questions, 8 provided qualitative input in all three, and the remaining participants responded “no need to add anything” in at least one or two of the qualitative questions. Saturation procedures were not applied, as all participant contributions were considered in the analysis.

#### 3.4.1. Knowledge

On the one hand, some of the suggestions were grouped under the category “Prior knowledge in the area of health” without creating a new category, as they were part of the specific knowledge within the area of health. In this sense, aspects such as knowledge of the Family and Community Health Model, knowledge of recent programs and strategies implemented in PHC and the use of computer systems for clinical and administrative management, experience in cross-cutting programs and topics such as social determinants and the municipal role were considered within this category.

Two new competencies of knowledge were identified that were not present in the original proposal. The first, “Knowledge of the regulatory and legislative framework of the PHC”, was based on suggestions regarding specific regulations as well as the internal regulations of establishments and the labour code. The second new category corresponds to “Knowledge of quality management and accreditation”, in response to explicit mentions of the PHC accreditation system, bidding processes, and quality standards. Finally, content related to planning and leadership was moved to the skills categories, and aspects such as professionalism or service orientation were moved to attitudes.

#### 3.4.2. Skills

The so-called “soft skills”, mentioned repeatedly, were reorganized into existing categories such as effective communication, empathy, self-criticism and conflict management. “Emotional intelligence” was added based on one of the suggestions. The concept of “institutional support” was also incorporated, referring to the need for managers to support teams in situations of pressure or disagreement with users. The creation of a new skill called “team well-being management” was also proposed, which groups together contribute to the prevention of burnout, the promotion of healthy environments, and the care of mental health.

#### 3.4.3. Attitudes

Fairness was integrated into neutrality, while humility and acknowledging mistakes were grouped under self-criticism. References to public service vocation were treated as cross-cutting themes, not observable attitudes. Two new attitudes were added: a positive and proactive attitude, reflecting commitment to improvement and problem-solving; and territorial management, highlighting awareness of the political and social context in which management takes place.

## 4. Discussion

This study validates a competency profile for PHC managers in Chile, contributing to a contextualized approach that strengthens local leadership.

The results showed a strong expert consensus supporting the proposal, indicating that the previous systematic review obtained accurate results that translate to the Chilean reality. Knowledge of health management, use of indicators, and management of the healthcare network were highly valued, which is consistent with studies that highlight the need for managerial skills to optimize decision-making in PHC [18,37]. These competencies are essential for addressing the challenges of system fragmentation, implementing integrated models, and ensuring resource efficiency [38]. An important aspect that requires clarification is the role of patient safety and quality. In the qualitative analysis, this theme did appear in the open-ended responses; however, it did not consolidate as an autonomous competency. During the categorisation process, contributions explicitly stating “Knowledge of the Family Health Model and Quality and Safety of health care” were integrated into the broader domain of “previous experience/knowledge in health/PHC”. This decision was based on the phrasing of the responses, which referred to knowledge of care models and quality/safety principles rather than to an explicitly defined managerial competency. For this reason, the content was categorised as part of prior experience in PHC instead of being treated as a separate domain. However, considering the Global Patient Safety Action Plan 2021–2030 [39], which highlights the need to strengthen and explicitly define patient safety competencies within primary health care; although these contributions were integrated into a broader category, they underscore an area that could warrants particular attention.

In addition, the experts considered it necessary to include knowledge about the regulatory framework and quality management and accreditation (based in two and four qualitative responses, respectively, Table A2). This is key for managers to make good decisions, use public resources well, and comply with health policies. Furthermore, quality management becomes very important knowledge, as it helps to deliver people-cantered, efficient and safe care. In this way, accreditation is not only a formal requirement, but also an opportunity to improve the functioning of the centre, which requires managers who are prepared to lead, work as a team and integrate the community into the process [40]. Emotional intelligence, institutional support, and well-being management skills were also considered. These competencies are justified by their close relationship with team sustainability, as they enable effective management of interpersonal dynamics, maintain group cohesion, and foster a healthy work environment. Creating an emotionally safe work environment is intrinsically linked to team well-being management, as fostering a supportive psychosocial climate allows employees to feel secure, resilient, and engaged, while mitigating stress and burnout and promoting overall mental health and job satisfaction [41,42]. In line with contemporary approaches to leadership, it is recognized that the workplace should be perceived as an emotionally safe environment, where the manager not only administers, but also acts as a facilitator of human and professional development [3,43]. It is important to highlight that “institutional support” was classified as a managerial skill rather than an organizational attribute. This distinction is grounded in the expert feedback, which framed the construct in terms of active managerial behaviours, protecting, validating, and supporting staff, particularly in situations of conflict or pressure with users. Conceptualizing it as a skill emphasizes that these behaviours are within the manager’s control and can directly influence team well-being, cohesion, and performance, independently of formal institutional policies or resources. This perspective aligns with contemporary leadership research, which identifies supportive managerial actions as key determinants of staff motivation, resilience, and effectiveness [3,43].

Finally, positive and proactive attitudes, as well as territorial management, were added. Their inclusion demonstrates a strategic approach that recognizes the value of situated leadership, capable of interpreting complex contexts and promoting relevant and participatory responses. In this sense, the explicit inclusion of these values in the profile transcends a merely technical or bureaucratic view of leadership, recognizing that community trust, institutional legitimacy and commitment to collective well-being are as essential as operational and management skills [44]. In addition, the manager’s proactive attitudes could serve as an example for the rest of the team, improving performance [2].

A central contribution of this study is the identification and integration of emotional and cognitive competencies as essential components of effective leadership in PHC management. Beyond technical and procedural knowledge, the inclusion of emotional intelligence, team well-being management, and institutional support reflects the critical role of affective capacities in sustaining team cohesion, preventing burnout, and fostering resilient work environments. Similarly, cognitive competencies such as understanding the regulatory and legislative framework and quality management and accreditation enable adaptive decision-making, strategic planning, and efficient problem-solving under complex, high-demand conditions. The incorporation of these new competencies aligns with the growing evidence that effective leadership involves the integration of executive, emotional, and social brain networks [2]. For example, meta-analytic evidence shows that executive functions such as inhibition, working memory and cognitive flexibility are positively correlated with empathy and related social cognitive processes (r = 0.20) [45]. Similarly, emotional intelligence has been shown via meta-analysis to correlate with employee outcomes (e.g., job performance, reduced stress) [46,47]. By explicitly incorporating these emotional and cognitive dimensions, the study emphasizes that effective leadership in PHC is not solely defined by managerial expertise, but by the ability to integrate executive functions and affective regulation, supporting both optimal organizational performance and the well-being of health teams [3,44].

In addition, when comparing the results with the international competency framework, such as that proposed by WHO-ASPHER for Europe [48], there is clear convergence in key areas such as leadership, resource management, teamwork, communication, and ethical practice. However, the profile obtained incorporates differential nuances that respond to the specificities of PHC and the organization of the health system in Chile, such as territorial management, team well-being, neutrality in decision-making, and motivation as a central attitude. These competencies emerge as necessary in contexts where local governance, social inequalities, and healthcare pressure have a decisive influence. In this sense, the novelty of the study lies not only in describing an ideal profile, but also in offering a benchmark adapted to the Latin American context that can guide appointment and professional development policies. Finally, many PHC managers lack formal management training, underscoring the need for programs that equip them to meet current challenges. This finding reinforces the relevance of integrating competency-based education models into professional training and postgraduate programs in health sciences, promoting leadership development tailored to PHC contexts.

Finally, although this profile is a solid starting point, its implementation within the Chilean PHC system may face several contextual and structural barriers. The persistent fragmentation and segmentation of the health system, along with social inequality, precarious living conditions, and increasing migratory flows, create heterogeneous service demands that complicate the standardization of managerial competencies across territories [30,31]. Resource limitations, high administrative burden, and variability in local governance may further restrict managers’ ability to incorporate new competencies into daily practice. Unequal access to training, supervision, and technological infrastructure, particularly in rural or underserved municipalities, may also result in uneven adoption of the benchmark. For these reasons, the profile should be viewed as a flexible rather than a static tool, allowing adaptation to the circumstances and capacities of each PHC centre to ensure its feasibility and relevance [30,31].

### Limitations and Future Lines of Research

This study has several limitations that should be acknowledged. The sampling strategy employed (convenience and snowball sampling) may have introduced selection bias and limits the generalisation of the results. Although the required number of experts was reached, greater representation from the south is recommended to improve territorial balance. Moreover, the central macro-zone was overrepresented (26 out of 36 participants), which reduces the extent to which the proposed competency profile can be extrapolated to the northern and southern macro-zones, given their distinct socio-geographical and organisational contexts. These limitations should be considered when interpreting the findings.

This imbalance is also consistent with the structural centralization of the Chilean health system, where most PHC managerial positions and training centres are in the central macrozone. Given regional differences, the profile should remain adaptable to local contexts. The validation focused on content adequacy, fulfilling the study’s aim, but further research should assess the profile’s practical use in selection, evaluation, and training of PHC managers. Despite the high expert consensus, external validation is still needed including pilot applications in selection and training processes, as well as longitudinal studies to explore implementation across diverse PHC settings and identify limitations. Furthermore, the use of non-probability sampling could have introduced selection bias, as participation depended on the availability and interest of the experts. Likewise, the assessments could have been affected by social desirability bias, leading some participants to evaluate the competencies more positively than they actually considered them to be. Finally, the absence of a second round of validation limited the possibility of re-evaluating items after receiving group feedback, which would have allowed for further refinement of the level of consensus. Future competency development and training initiatives could benefit from integrating insights from cognitive and affective neuroscience into leadership education. Neurocognitive assessment tools, simulation-based learning, and interventions targeting executive function and emotional regulation could enhance managers’ decision-making, adaptability, and team performance by strengthening neural mechanisms underlying self-regulation, empathy, and social cognition in complex health care environments. The resulting set of competencies provides a solid foundation upon which a more comprehensive and structured competency framework may be developed in future research.

## 5. Conclusions

A competency profile for PHC managers in Chile was validated through expert consensus. This profile integrates technical knowledge, managerial and leadership skills, and attitudes aimed at transforming health services. Key competencies include the ability to coordinate care networks, manage human resources, promote team well-being, lead ethically and adaptively, and understand territorial contexts as strategic components. Its methodological rigor and alignment with current health system challenges underscore its value as both a training and management tool. The profile holds strong potential to strengthen local leadership capacities, improve decision-making in PHC, and support the advancement of public policies rooted in the principles of collective health. In addition, this validated profile provides a valuable reference for competency-based education and training initiatives aimed at developing leadership and management capacities among PHC professionals. The profile is intended primarily as a national reference to guide PHC managerial development. At the same time, its structure is flexible enough to support local adoption by health services and adaptation by institutions. It may also inform competency-based training initiatives and internal promotion processes within the Chilean PHC system.

## Figures and Tables

**Table 1:** Shapiro–Wilk normality analysis.

	Age	Years of Experience in PHC	Years of Experience in Current Position	Years of Experience at Current Institution
North	0.728 *	0.763 *	0.877	0.895
Centre	0.951	0.922 *	0.919 *	0.973
South	0.863	0.860	0.869	0.869

* *p* < 0.05.

**Table 2:** Descriptive analysis.

	North (*n* = 5) Arica to Coquimbo	Centre (*n* = 26) Valparaíso to Biobío	South (*n* = 5) La Araucanía to Magallanes
Age M (SD)/Mn (Q1–Q3)	35 (32–35)	39.89 (7.20)	37.40 (4.62)
Gender (Women/Men)	4/1 (80%/20%)	15/11 (57.69%/42.31%)	3/2 (60%/40%)
Position			
Care role in primary care/teacher/researcher	0 (0%)	1 (3.85%)	0 (0%)
Care role in primary care/teacher	0 (0%)	6 (23.08%)	0 (0%)
Care role in primary care	3 (60%)	9 (34.62%)	4 (80%)
Manager in primary care	0 (0%)	8 (30.77%)	1 (20%)
Manager in primary care/care role in primary care	2 (40%)	2 (7.69%)	0 (0%)
Educational Level			
Doctorate	0 (0%)	1 (3.85%)	0 (0%)
Master’s degree	1 (20%)	13 (50%)	3 (60%)
Diploma	3 (60%)	11 (42.31%)	2 (40%)
Bachelor’s degree	1 (20%)	1 (3.85%)	0 (0%)
Years of experience in PHC M (SD)/Mn (Q1–Q3)	9 (9–11)	12 (12–15)	12.80 (4.49)
Years of experience at the current position M (SD)/Mn (Q1–Q3)	9 (8)	6.50 (4–10)	9.80 (4.44)
Years of experience in the institution M (SD)/Mn (Q1–Q3)	9.20 (7.92)	10.15 (4.31)	9.80 (4.44)
Competency training (yes/no/don’t remember)	3 (60%)/2 (40%)/0 (0%)	24 (92.31%)/1 (3.85%)/1 (3.85%)	5 (100%)/0 (0%)/0 (0%)

**Table 3:** CVI analysis.

Knowledge (6 Competences)		Skills (20 Competences)		Attitudes (14 Competences)	
1		0.97		0.99	
Previous in health/aps	1	Leadership	1	Autonomy	1
Healthcare network	1	Teamwork	1	Resilience	1
Health administration and management	1	Crisis management	0.94	Neutrality	1
Management and use of indicators	1	Institutional alignment	0.97	Coping skills	0.97
Health personnel management	0.97	Strategic vision	0.94	Creativity	0.97
Workflows and processes	1	Effective communication	1	Motivation	1
		Planning, monitoring and evaluation	1	Flexibility	1
		Recognising skills in others	0.94	Professionalism	1
		Conflict management	0.97	Proactivity	1
		Negotiation	0.97	Empathy	1
		Delegation	1	Professional self-realisation	1
		Decision-making	1	Acting ethically and responsibly	0.97
		Articulation skills	1	Self-criticism	1
		Change management	0.97	Initiative	1
		Persuasion	0.94		
		Relationship building	0.94		
		Analytical thinking	0.97		
		Innovative thinking	0.92		
		Performance evaluation	0.97		
		Team building	0.94		

## Data Availability

The data presented in this study are available on request from the corresponding author due to ethical and privacy restrictions.

## Appendix A

**Table A1:** Comparison between initial and final competency profile.

Knowledge	Skills	Attitudes
**Initial competencies**		
Previous in health/PHC	Leadership	Autonomy
Healthcare network	Teamwork	Resilience
Health administration and management	Crisis management	Neutrality
Management and use of indicators	Institutional alignment	Coping skills
Health personnel management	Strategic vision	Creativity
Workflows and processes	Effective communication	Motivation
	Planning, monitoring and evaluation	Flexibility
	Recognising skills in others	Professionalism
	Conflict management	Proactivity
	Negotiation	Empathy
	Delegation	Professional self-realisation
	Decision-making	Acting ethically and responsibly
	Articulation skills	Self-criticism
	Change management	Initiative
	Persuasion	
	Relationship building	
	Analytical thinking	
	Innovative thinking	
	Performance evaluation	
	Team building	
**Added competencies**		
Regulatory and legislative framework for PHC	Institutional support	Positive and proactive attitude
Quality management and accreditation	Team well-being management	Territorial management
	Emotional Intelligence	

## Appendix B

Final Competency Profile.

### Appendix B.1. Knowledge

1. Previous in health/PHC: Understanding of the principles, structures, and practices related to health promotion, prevention, and comprehensive care at the community and family levels.
2. Healthcare network: In-depth understanding of how the health system operates and how the different levels of care (primary, secondary, tertiary) are integrated, as well as the processes that ensure continuity and efficiency in patient care.
3. Health administration and management: Understanding principles and practices for organizing, directing, and coordinating human, financial, technological, and material resources within the health system to ensure effective and efficient service delivery.
4. Management and use of indicators: Ability to identify, analyse, and apply key metrics to assess service performance, quality of care, and population health outcomes.
5. Health personnel management: Competencies required to effectively manage human resources in the health sector, particularly in PHC, ensuring that teams operate efficiently and in line with strategic objectives.
6. Workflows and processes: Understanding and ability to manage, design, and optimize the sequence of activities and operations within a health organization or system, with a focus on PHC.
7. Regulatory and legislative framework for PHC: Ability to understand, interpret, and apply the laws, policies, and regulations governing Primary Health Care.
8. Quality management and accreditation: Conceptual and practical understanding of principles, tools, and processes associated with continuous quality improvement in health services.

### Appendix B.2. Skills

1. Leadership: Ability to influence, motivate, and guide others toward shared goals—not limited to formal authority, but also including positive influence and inspiring commitment.
2. Teamwork: Capacity to collaborate effectively with individuals of diverse skills and backgrounds, coordinating efforts and responsibilities to achieve common goals.
3. Crisis management: Set of actions and strategies to anticipate, address, and resolve critical situations that may endanger an organisation or system, requiring rapid and effective responses.
4. Institutional alignment: The extent to which an organization’s goals, strategies, values, and actions are aligned, ensuring coherence across departments and personnel.
5. Strategic vision: Ability to anticipate and plan by identifying long-term objectives, emerging trends, and necessary changes, guiding present actions toward sustainable success.
6. Effective communication: Skill to convey messages clearly and accurately, ensuring mutual understanding and facilitating agreements and actions—includes verbal and non-verbal communication.
7. Planning, monitoring, and evaluation: Ability to define objectives, set goals and deadlines, assign tasks, monitor progress, and evaluate outcomes to ensure successful implementation.
8. Recognising skills in others: Ability to identify and value the strengths and talents of others, assigning responsibilities or guiding development accordingly.
9. Conflict management: Skill to identify, address, and resolve disputes constructively, understanding root causes and maintaining relationships while promoting performance.
10. Negotiation: Process through which parties with shared or conflicting interests reach mutually beneficial agreements. It is key to problem-solving and achieving organisational goals.
11. Delegation: Ability to assign responsibilities to others effectively, granting the necessary authority and resources while maintaining adequate supervision.
12. Decision-making: Capacity to choose the best course of action among alternatives by weighing risks, benefits, and consequences in the short and long term.
13. Coordination skills: Ability to integrate and align different services, care levels, stakeholders, and resources to ensure comprehensive, continuous, and accessible care.
14. Change management: Skill to lead and support organisational transitions, addressing resistance, maintaining motivation, and achieving desired outcomes with minimal negative impact.
15. Persuasion: Ability to influence ideas or behaviours through logical argumentation and emotional connection, building trust and credibility.
16. Relationship building: Ability to establish and maintain positive, productive relationships with colleagues, supervisors, clients, and other stakeholders.
17. Analytical thinking: Ability to break down complex issues into components, evaluate information, and draw logical conclusions using critical and systematic reasoning.
18. Innovative thinking: Ability to generate new ideas and approaches, challenge conventional thinking, and propose creative solutions that add value.
19. Performance evaluation: Ability to assess individual or team performance objectively, based on clear criteria, and provide constructive feedback for improvement.
20. Team building: Ability to select, organize, and motivate team members to work collaboratively and efficiently toward common objectives.
21. Emotional intelligence: Ability to recognize, understand, and manage one’s own emotions and those of others, fostering effective relationships and a healthy work environment.
22. Institutional support: The managers’ role in backing their team during pressure or conflict, especially when staff act according to regulations.
23. Team well-being management: Ability to promote healthy work conditions, detect signs of stress or burnout, and implement preventive strategies to maintain team well-being.

### Appendix B.3. Attitudes

1. Autonomy: Willingness to make decisions and act independently, with self-regulation and responsibility in fulfilling tasks.
2. Resilience: Ability to adapt to and recover from adversity, learning and growing from challenges without lasting harm.
3. Neutrality: Attitude of impartiality in conflict resolution and decision-making, relying on facts and avoiding personal bias.
4. Coping skills: Proactive and effective approach to dealing with challenges, maintaining emotional control and seeking constructive solutions.
5. Creativity: Disposition to generate new ideas and approaches to improve processes and decision-making.
6. Motivation: Internal drive to pursue goals and achieve fulfilment, influencing engagement and persistence at work.
7. Flexibility: Readiness to adapt to changes or new demands with agility and effectiveness.
8. Professionalism: Commitment to ethical, respectful, and responsible behaviour, aligned with the expectations of the professional role.
9. Proactivity: Tendency to anticipate situations, take initiative, and seek improvement without waiting for instructions.
10. Empathy: Active disposition to understand and connect with others’ feelings and perspectives, showing genuine interest and sensitivity.
11. Professional self-realisation: Commitment to personal growth, striving for satisfaction and meaning in work through development and purpose.
12. Acting ethically and responsibly: Commitment to integrity, justice, and community welfare, with awareness of the impact of one’s actions.
13. Self-criticism: Willingness to evaluate oneself honestly, acknowledging strengths and areas for improvement to foster continuous learning.
14. Initiative: Internal drive to act independently, recognize opportunities, and take responsibility without external prompting.
15. Positive and proactive attitude: Orientation toward continuous improvement, problem-solving, and constructive engagement.
16. Territorial management: Ability to act with awareness of the political and social context of the local territory, aligning institutional management with local realities and PHC strategies.

## Appendix C

**Table A2:** Categorization of expert responses for knowledge.

Decision	Competence (Final)	Expert Statements (Verbatim, Translated)
Not added	—	“No” (33.33%, 12 of 36 responses)
Reorganized	Previous in health/PHC	“Knowledge of the Family Health Model and Quality and Safety of health care”; “Having knowledge of newly implemented strategies such as ECICEP”; “Health information systems or digital platforms”; “experience and cross-cutting knowledge of programs”; “Social determinants”; “Knowledge of the municipal role in PHC”; “Knowing all the possible programs that can be carried out in PHC in order to expand the portfolio of services of the facility (e.g., Vida Sana, Más AMA, Friendly Spaces, etc.)”; “Family Medicine and knowledge of the family and community health model. Health information systems or digital platforms. In terms of service delivery, some level of knowledge in chronic disease management and, in my opinion, it would be desirable to have basic training in the management of institutions for older adults.”
Added	Regulatory and legislative framework for PHC	“Knowledge of Laws 19,378, 18,883, 21,188, 21,643, 16,744, 20,545, 20,609, the Labor Code, the local regulation of the civil service career and the internal regulations”; “health legislative framework”
Added	Quality management and accreditation	“Knowledge in quality management and accreditation”; “Knowledge about public bidding processes”; “Knowledge of the PHC accreditation system”; “Knowledge of quality and accreditation”
Reallocated → Skills	Planning, monitoring and evaluation/Effective communication	“Health planning and programming, and strategic management of health information and communication”
Reallocated → Skills	Leadership	“Competencies and knowledge in organizational leadership”
Reallocated → Attitudes	Professionalism	“A manager must guarantee the delivery of a comprehensive, quality, and timely health service”

**Table A3:** Categorization of expert responses for skills.

Decision	Competence (Final)	Expert statements (Verbatim, Translated)
Not added	—	“No” (63.89%, 23 of 36 responses)
Reorganized	Existing categories (Effective communication, empathy, self-criticism, conflict management)	“Soft skills such as empathy, emotional intelligence, resilience, tolerance to stress and frustration, among others”; “Active and empathetic listening”
Added	Emotional intelligence	“Soft skills such as empathy, emotional intelligence, resilience, tolerance to stress and frustration, among others”
Added	Institutional support	“Empathy to understand the difficulties people face and the problems they encounter, since many times their work or conflicts are minimized, especially frontline staff dealing with demanding users. I don’t know how to call the skill of backing up staff when they give a correct instruction to a user and the user does not accept it (to prevent the staff member from feeling unprotected, without support).”
Added	Team well-being management	“Promoting good practices, knowledge and skill in caring for the mental health of health workers, prevention of burnout, flexibility in decision-making.”

**Table A4:** Categorization of expert responses for attitudes.

Decision	Competence (Final)	Expert Statements (Verbatim, Translated)
Not added	—	“No” (72.22%, 26 of 36 responses)
Refined	Neutrality	“Fairness in the treatment of staff”
Refined	Self-criticism	“Being consistent, humility in recognizing when they have been wrong”
Not added	—	“Public service vocation does not constitute an observable skill…”
Added	Positive and proactive attitude	“Positivism”; “Positive and proactive attitude”
Added	Territorial management	“I would add strategic territorial thinking”
